# New insights into protein-protein interaction data lead to increased estimates of the *S. cerevisiae *interactome size

**DOI:** 10.1186/1471-2105-11-605

**Published:** 2010-12-21

**Authors:** Laure Sambourg, Nicolas Thierry-Mieg

**Affiliations:** 1 Laboratoire TIMC-IMAG, BCM, CNRS UMR5525, Faculté de médecine, 38706 La Tronche cedex, France

## Abstract

**Background:**

As protein interactions mediate most cellular mechanisms, protein-protein interaction networks are essential in the study of cellular processes. Consequently, several large-scale interactome mapping projects have been undertaken, and protein-protein interactions are being distilled into databases through literature curation; yet protein-protein interaction data are still far from comprehensive, even in the model organism *Saccharomyces cerevisiae*. Estimating the interactome size is important for evaluating the completeness of current datasets, in order to measure the remaining efforts that are required.

**Results:**

We examined the yeast interactome from a new perspective, by taking into account how thoroughly proteins have been studied. We discovered that the set of literature-curated protein-protein interactions is qualitatively different when restricted to proteins that have received extensive attention from the scientific community. In particular, these interactions are less often supported by yeast two-hybrid, and more often by more complex experiments such as biochemical activity assays. Our analysis showed that high-throughput and literature-curated interactome datasets are more correlated than commonly assumed, but that this bias can be corrected for by focusing on well-studied proteins. We thus propose a simple and reliable method to estimate the size of an interactome, combining literature-curated data involving well-studied proteins with high-throughput data. It yields an estimate of at least 37, 600 direct physical protein-protein interactions in *S. cerevisiae*.

**Conclusions:**

Our method leads to higher and more accurate estimates of the interactome size, as it accounts for interactions that are genuine yet difficult to detect with commonly-used experimental assays. This shows that we are even further from completing the yeast interactome map than previously expected.

## Background

As the chief actors within the cell, proteins participate in every cellular process, from metabolism to mechanical structure, immune system or signaling pathways. To successfully fulfill their role, they stably or transiently interact with each other, forming a complex protein interaction network, or interactome. Thus, the comprehensive mapping and deciphering of theses interactomes is a prerequisite for the full understanding of any cellular system. Furthermore, interactomes can be used to infer the function and regulation of novel proteins (*e.g*. Tarassov *et al*. predict that the previously uncharacterized proteins YML018C, YMR221C and YDR119W are involved in autophagy [1]). However, when trying to extract information from protein interaction networks, one must be aware that they are far from comprehensive. Estimating the size of an interactome provides insight into the biological relevance of the conclusions drawn. For example, partial sampling from networks presenting a variety of degree distributions can result in apparent scale-free subnetworks, irrespective of the initial network's topology [2]: topology analyses based on incomplete data may not be valid. Moreover, the number of protein-protein interactions is an important parameter for evaluating the completeness of databases and current high-throughput experiments, in order to measure the remaining efforts and build a framework for future experiments [3,4]. We focus here on *S*. *cerevisiae*, one of the most studied eukaryotic model organisms and a widely-used test platform for new experimental techniques, in particular for protein-protein interaction (PPI) detection methods.

## Available data

The available datasets of protein-protein interactions fall into two categories: literature-curated (LC) and high-throughput (HT). LC data reports manually curated interactions described in the literature, usually obtained by low-throughput experiments [5]. While high-throughput datasets are typically produced by testing all pairs of proteins within a subspace determined solely by the availability of reagents, low-throughput experiments are often hypothesis-driven, for example targeted at proteins involved in a disease or in a particular cellular function. Additionally, both LC and HT data can be of different nature: some assays identify proteins that belong to the same complexes, and find mainly stable but potentially indirect interactions (*e.g*. Affinity purification followed by mass spectrometry [6,7]), while others such as HT-Y2H (high-throughput yeast two-hybrid [8-10]) or PCA (protein complementation assay [1]) search essentially for direct binary interactions that may be transient [11]. Finally, synthetic lethality, genetic suppression and genetic enhancement are examples of genetic interactions, which occur at the phenotypic level and rarely correspond to physical interactions [12]. In this study, we focus on direct binary physical interactions.

Any dataset may contain errors, and particular attention must be paid to false positives (proteins erroneously annotated as interacting). Since interacting proteins in Y2H are not expressed in their natural cellular context, false positives are restricted here to 'technical' false positives that are due to stochastic or systematic detection method artifacts, and we ignore 'biological' false positives where an interaction is indeed physically possible but not biologically relevant (*e.g*. if the proteins are never expressed in the same cellular compartment).

## Existing estimates

Since the publication of the first HT-Y2H datasets, several methods for estimating the size of the *S*. *cerevisiae *interactome have been proposed [5,10,13-18]; it is typically inferred to contain around 20,000 binary interactions, with extreme estimates ranging from 10,000 to 30,500. These methods are often based on analyses of the HT-Y2H genome-wide screens of the yeast interactome [8-10], and can be broadly divided into two categories. A first class involves the study of the overlap between two or more datasets [14-16,19], usually assumed to follow a hypergeometric distribution. Conceptually these methods differ mainly in their choice of datasets and estimations of error-rates. The second class of methods focuses on a single dataset. Two such methods [5,13] are based on an extrapolation of the number of interactions in an HT [13] or LC [5] subnetwork to the total number of yeast proteins. Another approach applied in the paper reporting the latest HT-Y2H dataset [10] relies on the estimation of their assay's characteristics within a sophisticated framework [3]. This provides detailed information but requires intimate knowledge of the dataset and/or performing additional experiments, hence it may be difficult to accomplish outside the laboratory that produced the data. Finally, Huang and coworkers [17,18] adapted capture-recapture theory and applied it using Interaction Sequence Tag (IST) counts. This is an interesting approach but is only applicable to library-screen-based HT datasets where the number of IST hits is available (a single dataset [8] among those considered in this study). Other estimates based on affinity purification-mass spectrometry data [19] have been proposed but these count indirect interactions and, as this work focuses on the binary interactome, are not directly relevant.

To date, most studies have not explicitly and comprehensively taken into account both LC and HT data. One recent method [10] did use a 'positive reference set' derived from LC data to assess the 'assay sensitivity' of their Y2H assay, but this dataset represents only a small sample of the available LC interactions and is focused on high confidence rather than wide coverage. However, recent results demonstrate the radically different view that these data offer. For example, the correlation between centrality and lethality, established in 2001 (Jeong et al. [20]) and considered as a given since then, was based on Uetz [9] and LC [21] data; this correlation does not exist [10] in the *Y2H-Union *dataset (the union of the 3 genome-wide HT-Y2H library screening results [8-10], see Methods, Datasets). One possible explanation lies in the intrinsically different strategies underlying low-throughput and high-throughput data collection (hypothesis-driven versus systematic). Additionally, only Y2H and PCA have been applied in a high-throughput setting whereas a wide variety of detection methods have been used at low-throughput. Thus high-throughput and low-throughput experiments may have explored different subspaces of the interactome: these two data sources appear complementary, and current estimates of the interactome size are questionable because usually based exclusively on one or the other. Finally, LC data includes highly focused and thorough studies of particular proteins, which may have allowed the identification of some interactions that are intrinsically difficult to detect. This has also never been considered.

We propose here a method for estimating the size of an interactome. It is based on dataset overlap, but takes into account both HT and LC data, as well as interactions that are hard to detect by taking advantage of the extensive literature curation efforts undertaken at SGD (the Saccharomyces Genome Database [22]).

## Results

### Method overview

Our method is based on a comparison between low-throughput binary physical data curated from the literature (*LowBP-LC*, obtained from the BioGRID database after filtering), and a binary physical high-throughput dataset (*HT-Union*, the union of a PCA [1] and three HT-Y2H [8-10] datasets, see Methods). Assuming that HT interactions are randomly drawn within the interactome, and thus independently of their presence in *LowBP-LC*, allows to estimate the interactome size. Indeed, under this assumption, the number of true positive HT interactions included in *LowBP-LC *follows a hypergeometric distribution ℋ (*N*, *m*, *n*), with *N *the total number of genuine interactions, *m *the number of true positive *LowBP-LC *interactions and *n *the number of true positive HT interactions. Thus, given an estimation of the false-discovery rate (*FDR *= *FP*/(*TP *+ *FP *) with FP and TP the numbers of false positives and true positives, respectively) of each dataset, one can compute the number of genuine interactions in the whole interactome. This is the basis for most methods relying on the overlap between datasets [14-16,19].

**Figure 1:**
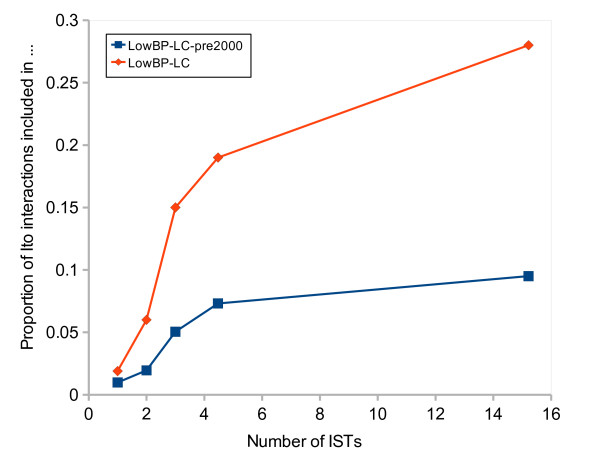
**Increased coverage by literature-curated datasets of interactions that are easier to detect by Y2H**. The proportion of Ito interactions present in *LowBP-LC *and in *LowBP-LC-pre2000 *(literature-curated interactions reported before 2000) is plotted as a function of the number of IST hits. Each point represents at least 200 interactions, and the number of IST hits is the weighted mean for these interactions.

However, all assays have their biases and limitations: some interactions may be easy to detect with one assay and difficult or impossible with another. In addition, most HT datasets were obtained with Y2H, but this assay is also widely used in low-throughput studies - it provides support for 53% of *LowBP-LC *interactions according to BioGrid evidence codes. It follows that *LowBP-LC *is expected to be enriched in interactions that are readily detectable with Y2H. This hypothesis is supported by studying Ito and co-workers' data [8]. Indeed, we used the number of IST hits (interaction sequence tags) for each interaction as an indicator of the difficulty to detect it: interactions with more ISTs are easier to detect, at least in Ito and coworkers' version of the Y2H protocol. We observed that the number of IST hits is clearly correlated with over-representation in *LowBP-LC *(See Figure 1 and Methods). As this phenomenon exists with both *LowBP-LC *and *LowBP-LC-pre2000 *(interactions reported before 2000), it is not due to the fact that low-throughput experiments could have been designed to confirm *Ito-Core *interactions (HT-Y2H interactions seen at least 3 times in Ito et al. [8], 2001). In addition, although the lower representation observed for interactions with 1 and 2 IST hits is likely partly due to higher FDRs among these interactions, reported as lower confidence in the original article [8], the coverage by *LowBP-LC *keeps increasing with the number of ISTs for interactions with 3 or more ISTs. These putative interactions - including any false positives among them - are well reproducible in this particular experimental system, hence the FDR is not expected to decrease when the number of ISTs increases. We conclude that the presence of an interaction in *LowBP-LC *is positively correlated with the ease of finding it by Y2H: *LowBP-LC *is indeed enriched in Y2H-strong interactions. Thus the assumption that HT and LC data are independent subsets of the complete interactome does not hold, and the simple dataset overlap method described above leads to underestimating the interactome size.

Our method can be summarized as follows. In order to alleviate this problem, we restrict the *LowBP-LC *dataset to interactions involving proteins that have been thoroughly studied: we show that these proteins have likely been subjected to a wider variety of assays, leading to a less biased view of the interactome. We then estimate the FDRs of *LowBP-LC *and of each HT dataset, using dataset overlap to relate the HT FDRs to one another. Finally, we model the number of HT true positives included in *LowBP-LC *restricted to well-studied proteins by a hypergeometric distribution ℋ (*N*, *m*', *n*), with *N *and *n *as described above and *m*' the number of true positive *LowBP-LC *interactions involving well-studied proteins (equation (5)). This leads to an estimation of the interactome size *N*.

### Taking into account how thoroughly proteins have been studied

**Figure 2:**
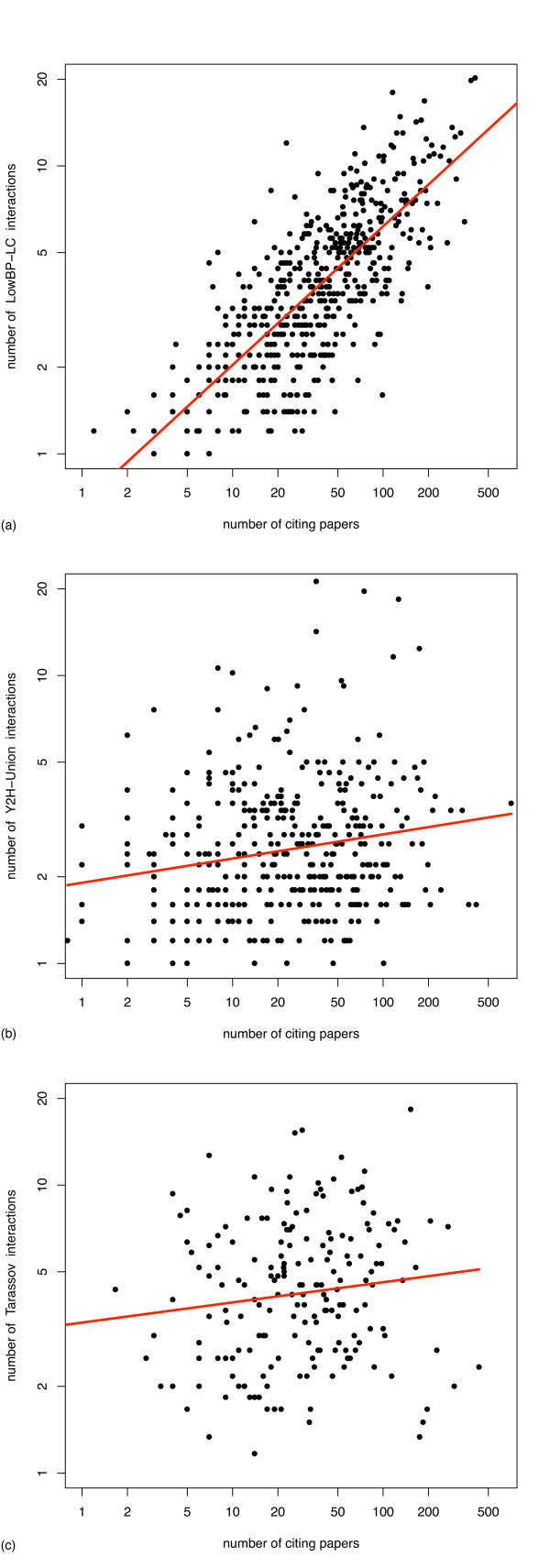
**Relation between the level of study and the degree of proteins in various datasets**. Log-log scale linear regression between the number of interactions (in the indicated dataset) involving a protein and the number of papers referencing that protein, using binned data (each point represents 5 proteins). (a) *LowBP-LC *interactions, *R*^2 ^= 0.59, P = 2 · 10^-103^, slope = 0.48. (b) *Y2H-Union *interactions, *R*^2 ^= 0.04, *P *= 1.0 · 10^-4^, slope = 0.08. (c) *Tarassov *interactions, *R*^2 ^= 0.01, *P *= 0.07, slope = 0.07.

We examined the relation between a protein's degree (*i.e*. the number of interactions it is involved in) and how thoroughly it has been studied, modeled as the number of papers in which the protein has been cited (according to the Saccharomyces Genome Database [23], see Methods). This revealed a strong correlation between these two quantities for the *LowBP-LC *dataset (Figure 2a): as expected, literature curation has reported many more interactions for highly studied proteins than for poorly studied ones. More surprisingly, a small but significant correlation also exists for the *Y2H-Union *dataset (Figure 2b). We see no reason why a proteome-wide Y2H screen would identify a larger proportion of the interactions that can be established by well-studied proteins, therefore this observation suggests that the density of the complete interactome is higher for well-studied proteins than for poorly studied ones. The statistical test is inconclusive with the *Tarassov *data (Figure 2c). Another unexpected observation is that even for well-studied proteins, *LowBP-LC *data are far from comprehensive: based on the available HT data for these proteins, we estimate the false negative rate (*FNR *= *FN*/(*TP *+ *FN*) with TP and FN the numbers of true positives and false negatives) of *LowBP-LC *restricted to well-studied proteins at approximately 60% (see Methods and Tables 1 and 2).

**Table 1:** Estimated false negative rate of LowBP-LC restricted to interactions involving well-studied proteins.

Well-studied cutoff	*Uetz-Screen*	*Ito-Core*	*CCSB-YI1*	*Y2H-Union*	*Tarassov*	*HT-Union*
100	0.64	0.66	0.66	0.68	0.63	0.67
105	0.63	0.65	0.65	0.67	0.61	0.66
110	0.64	0.66	0.65	0.67	0.61	0.66
115	0.65	0.66	0.64	0.67	0.61	0.66
120	0.66	0.65	0.63	0.66	0.62	0.65
125	0.66	0.66	0.65	0.67	0.61	0.66
130	0.63	0.49	0.63	0.63	0.62	0.63
135	0.60	0.49	0.62	0.61	0.62	0.62
140	0.60	0.47	0.62	0.61	0.64	0.62
145	0.62	0.45	0.61	0.60	0.64	0.61
150	0.63	0.45	0.61	0.61	0.64	0.62

The false negative rate is computed separately with each high-throughput dataset, using a cutoff to consider proteins well-studied ranging from 100 to 150 and a reference FDR for *CCSB-YI1 *set at 0.25.

**Table 2:** Influence of the CCSB-YI1 FDR on the LowBP-LC well-studied false negative rate.

*CCSB-YI1 *FDR	*Uetz-Screen*	*Ito-Core*	*CCSB-YI1*	*Y2H-Union*	*Tarassov*	*HT-Union*
0.15	0.67	0.66	0.65	0.68	0.57	0.66
0.25	0.63	0.62	0.61	0.64	0.53	0.62
0.35	0.59	0.57	0.57	0.58	0.48	0.56

The false negative rate of *LowBP-LC *restricted to interactions involving well-studied proteins is computed with the different datasets, when the *CCSB-YI1 *FDR ranges from 0.15 to 0.35, using a well-studied cutoff set at 125.

### Well-studied data comprise interactions that are difficult to detect

**Figure 3:**
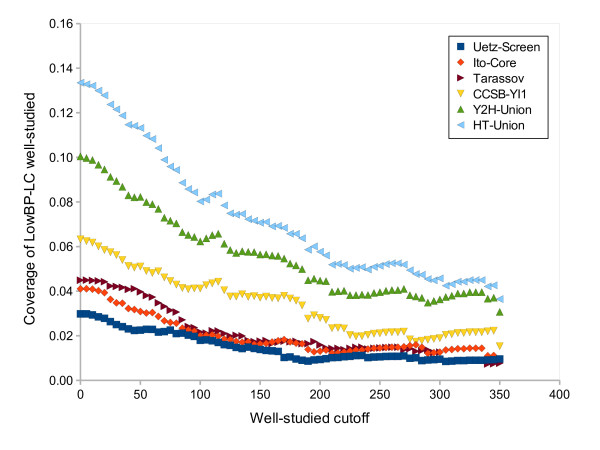
**Coverage of *LowBP-LC *well-studied by each high-throughput dataset**. The proportion of *LowBP-LC *interactions involving well-studied proteins that are covered by each HT dataset is plotted as a function of the 'well-studied cutoff', *i.e*. the minimum number of papers referencing a protein for it to be considered well-studied.

A closer look at the interaction data concerning well-studied proteins leads to another surprising discovery: HT data covers *LowBP-LC *much better than it does *LowBP-LC *restricted to interactions involving well-studied proteins (Figure 3). Note that this is not due to the fact that *LowBP-LC *has a better coverage of the complete interactome restricted to well-studied proteins: indeed, the completeness of *LowBP-LC *should not affect the proportion of its interactions that are present in an independent subset of the interactome. Thus, we see only two possible explanations.

First, this could be simply because the rate of false positives in *LowBP-LC *increases when restricting this dataset to well-studied proteins. Cusick et al. [24] recurated 100 literature-curated yeast interactions, which allows us to invalidate this hypothesis: for these interactions, we found that false positives are not over-represented among *LowBP-LC *interactions involving well-studied proteins (well-studied interactions represent 21.4% of the false positives and 22% of the true positives, see Methods).

As an alternative explanation, we propose that in-depth studies discover interactions that are difficult to detect by most widespread methods, hence are under-represented in HT datasets. To test this hypothesis, we examined whether the experimental methods used to demonstrate *LowBP-LC *well-studied interactions differed significantly from those used to demonstrate other *LowBP-LC *interactions, using the BioGrid experimental evidence codes. We observed that interactions in the well-studied subset are less frequently supported by Y2H (down 13.9% from 58.6% to 44.7%, p-value < 2.2e-16), while they are significantly more frequently supported by biochemical activity assays such as those detecting phosphorylation or ubiquitination (Biochemical Activity, up 12.4% from 11.1% to 23.5%, p-value < 2.2e-16), as well as in vitro assays using purified proteins (Reconstituted Complex, up 8.5% from 33.5% to 42%, p-value = 5.5e-12). Thus well-studied proteins have more often been subjected to labor-intensive interaction detection methods, which may allow the detection of a wider variety of interactions. To sum up, this supports the hypothesis that literature-curated interaction data involving well-studied proteins comprise interactions that, although genuine, are difficult or impossible to detect using labor-efficient methods such as Y2H.

Taking into account the level of study of proteins may thus allow to account for these interactions, hence lead to more accurate estimates of the size of an interactome.

### *LowBP-LC *false positives

Literature-curated data has been commonly assumed of excellent quality, but a recent study showed that curation errors may not be so infrequent. Cusick *et al*. [24] recurated 100 yeast interactions supported by a single paper, assigning a confidence score to each. They reported that 35% of these interactions were erroneous and that 40% could be not verified. For this study, we considered that among *LowBP-LC-Unique *(interactions from *LowBP-LC *supported by a single paper, and not found in the HT dataset), 35% were false positives. The initial report has been debated [25,26] and this may be an overestimate, which would result in our underestimating the interactome size. Interactions reported in more than one paper, or also detected by an HT experiment, were considered true positives.

### HT false positives

The initial mistrust of HT-Y2H assays was largely based on an analysis [27] benchmarking HT datasets against a set of protein complexes expanded with the matrix model, and does not seem relevant anymore [10,18]. Indeed, after the publication of the first HT-Y2H datasets, several methods estimated their FDRs at ~ 50% (*e.g*. [14,16]). However, by retesting their own data with orthogonal assays, Yu *et al*. [10] have estimated the FDR of *CCSB-YI1*, their proteome-wide HT-Y2H dataset, at 0-6%, and showed that *Uetz-Screen *(the Uetz *et al*. HT-Y2H library screening result [9]) and *Ito-Core *are also of high quality. Based on the capture/recapture method, Huang *et al*. [18] have evaluated the FDR of *Ito-Full *to 26%. *Ito-Full *is comprised of all interactions from Ito *et al*. [8] including those reported as low confidence in the original publication, and is known to have the lowest quality (*e.g*. [10,14,28]). As there is no consensus on the order of magnitude of these FDRs, we decided to apply our method with different FDR values. The *CCSB-YI1 *FDR is taken ranging from 15% to 35% and the other HT FDRs are computed as described below.

We developed a simple method for comparing the FDRs of high-throughput datasets, based on the hypothesis that the *LowBP-LC *coverage of HT true positives is the same for each HT dataset (see Methods). Under this assumption, we established a simple relation between the FDRs of HT datasets (Methods, equation (1)). However, if some low-throughput experiments were performed to verify interactions reported in high-throughput datasets, an important bias may favor older datasets, which will 'artificially' have more interactions in common with *LowBP-LC*. This problem can be addressed by restricting *LowBP-LC *to interactions reported before 2000 (the publication date of the oldest HT dataset), yielding another dataset called *LowBP-LC-pre2000*. In fact, *Ito-Core *and *Uetz-Screen *(published in 2001 and 2000) have a higher proportion of interactions in common with *LowBP-LC *than *CCSB-YI1 *(published in 2008), whereas with *LowBP-LC-pre2000*, the proportions are similar (Table 3). We therefore used *LowBP-LC-pre2000 *to estimate the HT FDRs. For example, assuming a *CCSB-YI1 *FDR of 25%, FDRs of Y2H datasets range from 15% to 25% (Table 4).

**Table 3:** Proportion of HT interactions included in LowBP-LC-pre2000 and LowBP-LC for the different datasets.

	*Uetz-Screen*	*Ito-Core*	*CCSB-YI1*	*Y2H-Union*	*Tarassov*	*Ito-Full*
*LowBP-LC-pre2000*	0.0831	0.0767	0.0734	0.0634	0.0264	0.0235
*LowBP-LC*	0.2017	0.2254	0.1617	0.1601	0.0746	0.0637

**Table 4:** Estimated false discovery rate of each high-throughput dataset.

	*CCSB-YI1*	*Uetz-Screen*	*Ito-Core*	*Tarassov*	*Ito-Full*
FDR	0.25	0.15	0.21	0.73	0.76

The FDRs are computed with eq (3), setting the *CCSB-YI1 *FDR at 0.25. As discussed, the FDR that can be computed for the *Tarassov *dataset is a rough upper bound.

Likewise, historical reasons may favor Y2H over PCA. Indeed, Y2H was proposed in 1989 [29], and has been widely used in low-throughput experiments, whereas PCA was first described in 2000 [30]. We cannot correct for this bias because restricting *LowBP-LC *to interactions reported before 1989 yields a very small dataset. As a consequence the FDR of 73% that can be computed for *Tarassov *(PPIs detected by high-throughput protein complementation assay [1]) may be largely overestimated and is only a rough upper bound.

### Estimating the interactome size

Starting with the number of *LowBP-LC *interactions involving well-studied proteins (2572 interactions), we removed the expected number of false positives (35% of *LowBP-LC-Unique*). We then calculated on the one hand the number of interactions, all considered as genuine, in the intersection between the *LowBP-LC *well-studied subset and the HT dataset (144 interactions for *HT-Union*, see Table 5 for the other datasets), and on the other hand the estimated number of true positives in the whole HT dataset, taking into account HT false positives by using the HT FDRs estimated as described above and assuming an FDR of 25% for *CCSB-YI1 *(~ 2814 true positives in *HT-Union*, see Table 5 for the other datasets). Taken together, this allows to estimate the size of the binary yeast interactome at ~ 37, 600 interactions (95% confidence interval: 32252-43472, constructed with the normal approximation method [31]). Details on the calculation are provided in Methods.

**Table 5:** Calculation steps leading to the interactome size. The well-studied cutoff is set at 125 papers and the CCSB-YI1 FDR at 0.25.

	*Uetz-Screen*	*Ito-Core*	*CCSB-YI1*	*Y2H-Union*	*Tarassov*	*HT-Union*
*LowBP-LC *well-studied size				2572		
*LowBP-LC *well-studied TPs	1905.95	1908.4	1911.55	1916.45	1909.45	1922.75
HT TPs	572.4	654.2	1349.3	2171.8	746.2	2814
HT∩*LowBP-LC *well-studied	30	35	72	112	38	144
Estimated size	36366	35670	35822	37163	37494	37574

**Figure 4:**
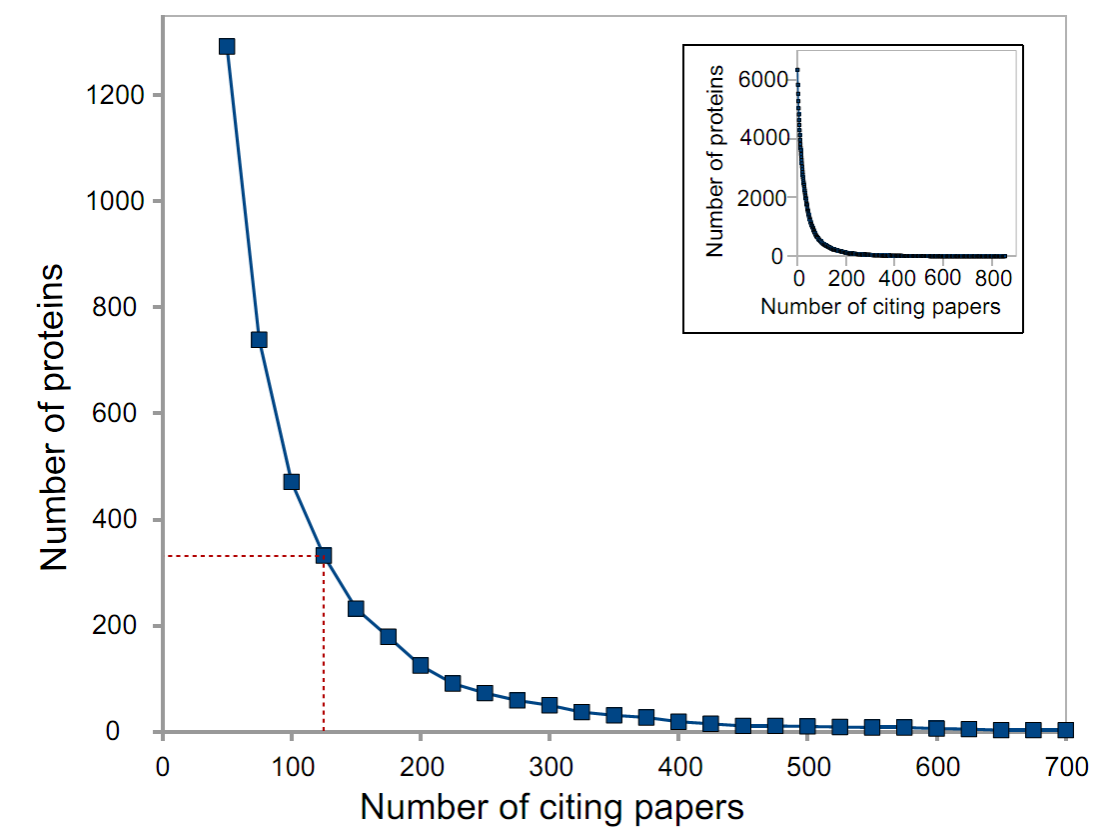
**Number of well-studied proteins**. The number of proteins in the well-studied subset is plotted as a function of the well-studied cutoff value. The main figure is restricted to proteins cited in at least 50 papers, while the inset shows the complete graph (starting at one paper). The well-studied cutoff value is the minimum number of papers referencing a protein, for this protein to be considered well-studied.

**Figure 5:**
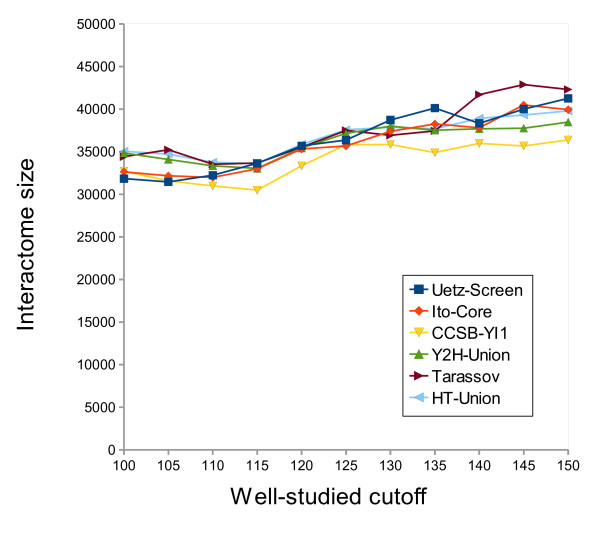
**Estimated size of the yeast interactome**. The predicted number of binary physical protein-protein interactions that can occur in *S. cerevisiae *is plotted as a function of the well-studied cutoff value, using each high-throughput dataset and a *CCSB-YI1 *FDR of 0.25. The well-studied cutoff value is the minimum number of papers referencing a protein, for this protein to be considered well-studied.

**Figure 6:**
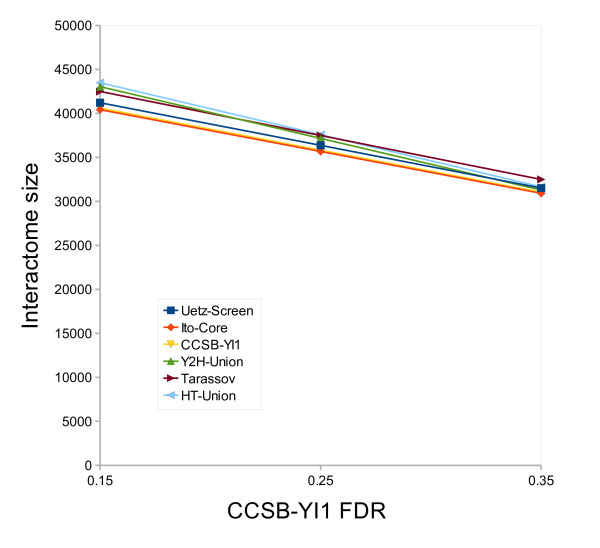
**Influence of the *CCSB-YI1 *FDR on the estimated interactome size**. The predicted size of the *S*. *cerevisiae *interactome is plotted using each high-throughput dataset, when the *CCSB-YI1 *FDR ranges from 0.15 to 0.35. The well-studied cutoff (number of papers for a protein to be considered well-studied) is set at 125 papers.

The *LowBP-LC *well-studied subset was defined with a cutoff (number of referencing papers for a protein to be considered well-studied) of 125 papers, which seems a good compromise between the number of proteins in the subset and how thoroughly they have been studied (Figure 4). The choice of this cutoff or even changes in the HT datasets have little influence on the estimate: it varies between 30,500 and 43,000 interactions, with a cutoff ranging from 100 to 150 and using all the different HT datasets, either singly or merged (Figure 5). Because of the *LowBP-LC */HT correlation, which is likely still present even when using the well-studied subset of *LowBP-LC*, the results presented here may be underestimated. Obviously, increasing the estimated HT FDRs decreases the interactome size (Figure 6), and more precise results could be obtained with better estimates of these FDRs.

By and large, our estimates are higher than previous ones, which is reasonable as we used all available datasets and took advantage of their complementarity, and we accounted for interactions that are difficult to detect.

## Discussion

As mentioned in the introduction, several methods based on dataset overlap have been proposed for estimating the yeast interactome size [14-16]. The main differences between these methods lie in the error-rate estimations and in the datasets used. While Grigoriev and co-workers [15] consider that false positives and false negatives compensate each other, d'Haeleseer and Church [16] estimate false-discovery rates thanks to the overlap of two HT datasets with a reference LC dataset, and Sprinzak and co-workers' FDR estimation [14] is based on co-localization data. In our method, a reference FDR for one dataset was chosen following a review of the literature, and the overlap between high-throughput and literature-curated data is used to derive the FDRs of other HT datasets from the reference FDR, somewhat similarly to d'Haeleseer and Church. Another important factor for this class of methods lies in the choice of datasets, beyond the necessity of selecting appropriate data (e.g. genetic interactions or co-complex membership may not be directly relevant when studying binary physical interactions). While considering only HT datasets [15] restricts the estimation to interactions that can be detected with the HT method, using a gold standard reference set that is assumed error-free [14,16] is also problematic. In our method carefully selected LC and HT data are combined, taking into account error-rate estimations for each dataset.

The main advantages of our method are the following. First and foremost, by leveraging the available knowledge of how extensively proteins have been studied, our method accounts for interactions that are genuine yet difficult to detect with commonly-used experimental assays. This significantly increases the predicted interactome size, and has never been taken into account. Secondly, it is applicable to any dataset or union of datasets, and it allows to use most of the available data independently of the experimental detection methods. Thus, the estimates are easy to update when new datasets become available. Furthermore, our model does not directly rely on a gold standard (*i.e*. a subset assumed to contain only true positives), which can be difficult to construct and can introduce biases of its own. Likewise, as no dataset is error-free, it is important to consider error rates of both HT and LC datasets.

We have also shown that well-studied proteins appear capable of establishing more interactions than poorly studied ones (Figure 2b). This probably stems from the fact that well connected proteins are more likely to play important roles in diverse cellular functions, and therefore attract more attention from the community. Our method inherently takes into account this bias. In addition, our method is robust with respect to the choice of HT datasets. Contrary to other estimates [13,14], which increase by 90% and 66% when substituting datasets (respectively *Ito-Full *for *Uetz *and *Uetz *for *Ito-Core*), ours only changes by at most 15% when using different Y2H datasets (at any given well-studied cutoff). Even when comparing estimates based on data obtained by very different assays (Y2H and PCA), the variation remains low (20%). Lastly, the results presented here are for *S. cerevisiae*, but our method could be applied to other organisms, as long as a genome-wide screen as well as significant literature curation have been performed. A potential weakness of our method is that it relies on overlap between datasets that can be small, which may affect the robustness of the estimates.

## Conclusion

In this work, we have analyzed HT and LC data while considering how thoroughly each protein has been studied. This has provided novel insight into existing interactome datasets: on the one hand, well-studied proteins seem capable of establishing more interactions than poorly studied ones, and on the other hand, in-depth studies of these well-studied proteins have allowed to identify interactions that are difficult to detect. Together with the combined use of LC and HT data, these observations allow to accurately estimate the interactome size. Our results show that the size of interactomes tend to be underestimated, as previous estimates are usually based on only one source of data and do not take into account interactions difficult to detect. No high-throughput technique can detect all interactions, and false negatives are unavoidable [32]. As a consequence, a variety of methods must be considered when working with interactome mapping, and new strategies such as prioritization and smart-pooling should be employed [4,33,34]. Extensive efforts will be required before an interactome map can be called 'complete', and until then biological conclusions based on the analysis of available data must be drawn with care.

## Methods

### Datasets

*LowBP-LC *contains 6, 272 low-throughput binary physical interactions gathered from BIOGRID-ORGANISM-Saccharomyces_cerevisiae-3.0.64.tab (downloaded from the BioGRID website) [35]. All papers referencing more than 100 interactions were considered as high-throughput, and their interactions were excluded. Among the remaining interactions, only binary physical data was kept, *i.e*. interactions whose detection method was by Reconstituted Complex, Two-hybrid, Far Western, Biochemical Activity, Co-crystal Structure, Protein-peptide, PCA or FRET (fluorescence resonance energy transfer).

*Ito-Core *[8], *Uetz-Screen *[9], *CCSB-YI1 *and *Y2H-Union *[10] are HT-Y2H datasets: *Ito-Core *contains the interactions seen at least 3 times by Ito *et al*., *Uetz-Screen *is the Uetz *et al*. genome-wide library screening result, and *Y2H-Union *is the union of these two datasets with *CCSB-YI1 *[10]. All these Y2H datasets were downloaded from the Center for Cancer Systems Biology website [36]. *Ito-Full *contains all interactions from Ito *et al*. [8]. It was downloaded from the Ito Laboratory website [37]. *Tarassov *are the PPIs detected by high-throughput protein complementation assay [1] (provided as supplementary material in the original publication). *HT-Union *contains all interactions from all HT datasets.

The level of study of a protein is modeled by the number of papers in which it has been cited, computed from a table of associations between literature and genes (downloaded from the Saccharomyces Genome Database [23] on 2010/05/03). Comparing HT FDRs requires to restrict the *LowBP-LC *dataset to interactions reported before 2000 in *LowBP-LC-pre2000*. *LowBP-LC-Unique *are interactions from *LowBP-LC *supported by a single paper, and not found in the considered HT dataset.

Additional file 1 presents the number of interactions and unique proteins in each dataset and intersection of datasets. All datasets are provided in Additional file 2.

### The false positive rate of *LowBP-LC *does not depend on the level of study

Cusick *et al*. recurated 100 literature-curated yeast interactions, assigning confidence score for each one: 0 for no confidence, 1 for low confidence or unsubstantiated and 2 for substantiated or high confidence. We therefore considered interactions with a score of 0 to be false positives, and those with a score of 2 to be true positives. We then computed the proportion of these interactions that involve well-studied proteins for each category. Among the 35 false positive interactions and the 25 true positives, respectively 21.4% and 22% involve a well-studied protein.

### *LowBP-LC *false negatives

Hypothesizing that HT well-studied and *LowBP-LC *well-studied are independent allows to estimate the expected number of genuine interactions involving well-studied proteins, and thus the *LowBP-LC *well-studied false negative rate:

$$FNR_{LowBP\text{-}LC_{WS}}=1-\frac{TP_{HT_{WS}\cap LowBP\text{-}LC}}{TP_{HT_{WS}}}$$

with $TP_{HT_{WS}}$ the estimated number of true positives in *HT_WS_*, the HT dataset restricted to interactions involving well-studied proteins, and $TP_{HT_{WS}\cap LowBP\text{-}LC}$ the number of true positives within the intersection between *HT_WS _*and *LowBP-LC*.

### A relation between HT FDRs

To decrease the potential correlation between *LowBP-LC *and older HT-Y2H datasets due to recent studies that could have been designed to confirm HT interactions, the *LowBP-LC *dataset used for the FDR calculations contains only interactions reported in publications published before 2000 (publication date of the oldest HT dataset). Consider two HT datasets, denoted 1 and 2 (*e.g. Ito-Core *and *CCSB-YI1 *), each partitioned into three subsets A, B and C, respectively the true positives included in *LowBP-LC-pre2000*, the true positives not included in *LowBP-LC-pre2000 *and the false positives. We consider that HT interactions also present in *LowBP-LC-pre2000 *are true positives (because detected by two independent methods). Therefore, *LowBP-LC-pre2000 *and C are disjoint. Hypothesizing that the proportion of true positive HT interactions in *LowBP-LC-pre2000 *is independent of the HT dataset yields:

$$\frac{A_{1}}{B_{1}}=\frac{A_{2}}{B_{2}}.$$

The proportion of HT interactions included in *LowBP-LC-pre2000 *(*A*/(*A *+ *B *+ *C*)) can be easily computed, and denoting α as

$$\frac{A_{1}}{A_{1}+B_{1}+C_{1}}=\alpha \cdot \frac{A_{2}}{A_{2}+B_{2}+C_{2}},$$

we obtain a relation between the false-discovery rates of the two datasets, defined as $FDR=\frac{C}{A+B+C}:$

(1) $$FDR_{1}=\alpha \cdot FDR_{2}+1-\alpha .$$

In the rest of this work, we always use *CCSB-YI1 *for set 2.

### Computing the interactome size

#### Parameters

• *HT *: the *HT *dataset used.

• *Well-studied cutoff*: number of papers referencing a protein to consider it well-studied.

• *FDR_Y I1 _*: the *CCSB-YI1 *FDR, required to compute the FDRs of other HT datasets.

#### Abbreviations and notations

• *WS*: well-studied.

• *TP_Dataset_*: estimated number of true positives in *Dataset*.

• |*Dataset*|: size of Dataset.

• *Is*: Interactome size.

#### HT true positives

• The FDR of *Ito-Core, Uetz-Screen *and *Tarassov *is calculated from the FDR of *CCSB-YI1 *as described in Methods, A relation between HT FDRs:

$$FDR_{HT}=\alpha \cdot FDR_{YI1}+1-\alpha$$

• The number of HT true positives is then computed as follows:

(2) $$TP_{HT}=\left|HT\right|-\sum \left|HT_{i}\right|*FDR_{HT_{i}}$$

where *HT_i _*iterates over the datasets making up HT for union datasets (*e.g*. for *Y2H-Union*: *Ito-Core*, *Uetz-Screen *and *CCSB-YI1 *), or HT itself for individual datasets such as *Ito-Core*.

#### LowBP-LC true positives

(3) $$\begin{array}{l} TP_{LowBP\text{-}LC_{\text{WS}}}=\\ \;\;|LowBP\text{-}LC_{\text{WS}}|-35\%\cdot \left|LowBP\text{-}LC\text{-}Unique_{\text{WS}}\right| \end{array}$$

Where *LowBP-LC-Unique_WS _*contains *LowBP-LC *interactions involving well-studied proteins, supported by a single paper and not in the HT dataset.

#### True positives in the intersection

All interactions in the intersection between HT and *LowBP-LC *are considered true positive, so:

(4) $$TP_{HT\cap LowBP\text{-}LC_{WS}}=\left|HT\cap LowBP\text{-}LC_{WS}\right|.$$

#### Interactome size

The hypergeometric assumption discussed in Results, Method overview leads to:

(5) $$Is=\frac{TP_{HT}\cdot TP_{LowBP\text{-}LC_{WS}}}{TP_{HT\cap LowBP\text{-}LC_{WS}}}$$

with *TP_HT _*, $TP_{LowBP\text{-}LC_{WS}}$ and $TP_{HT\cap LowBP\text{-}LC_{WS}}$ computed as described above (equations (2), (4) and (4)).

This can be expanded to:

$$Is=\frac{TP_{CCSB\text{-}YI1}\cdot TP_{LowBP\text{-}LC_{WS}}\cdot \left|HT\cap LowBP\text{-}LC\text{-}pre2000\right|}{\left|CCSB\text{-}YI1\cap LowBP\text{-}LC\text{-}pre2000\right|\cdot \left|HT\cap LowBP\text{-}LC_{WS}\right|}$$

This expanded form allows to study the influence of the various parameters. All relevant scripts are distributed under the GNU General Public License in Additional file 2.

### Presence of 'Y2H-strong' interactions in *LowBP-LC*

To examine whether interactions that are more easily detected in Y2H are also overrepresented in *LowBP-LC*, we gathered *Ito-Full *hits and binned them by increasing number of ISTs, each bin containing at least 200 interactions. Each bin is represented by the weighted mean of the number of ISTs, and the proportion of interactions present in *LowBP-LC*. In order not to separate interactions with the same number of ISTs, some bins (particularly single hits) are larger than others. This analysis is performed both with the complete *LowBP-LC *and with *LowBP-LC-pre2000 *(*LowBP-LC *interactions reported before 2000)(Figure 1).

## Abbreviations

PPI: protein-protein interaction; LC: literature-curated; HT: high-throughput; Y2H: yeast two-hybrid; PCA: protein complementation assay; FDR: false-discovery rate; IST: interaction sequence tag.

## Authors' contributions

NTM designed the study. LS implemented the method and performed the analyses. Both authors drafted and revised the manuscript. They have read and approved its final version.

## Supplementary Material

Additional file 1 **Number of interactions and proteins in each dataset**. Additional file 1 presents the number of interactions and unique proteins in each dataset and intersection of datasets. Click here for file

Additional file 2 **Datasets and scripts**. Additional file 2 is an archive that includes all scripts, distributed under an open source license, as well as all datasets used in this study. Click here for file
