# Supplementary material for: New insights into protein-protein interaction data lead to increased estimates of the S. cerevisiae interactome size
Source: BMC Bioinformatics. 2010 Dec 21;11:605. doi: 10.1186/1471-2105-11-605 (PMC3023808; doi:10.1186/1471-2105-11-605)

New insights into protein-protein interaction data lead to increased estimates of the *S. cerevisiae* interactome size - Additional file 1

Laure Sambourg and Nicolas Thierry-Mieg

Additional file 1 presents the number of interactions and unique proteins in each dataset and intersection of datasets.

I) Number of interactions and unique proteins in each dataset

Additional Table 1
Number of interactions and unique proteins, for each dataset used in the study.

Dataset name	Interactions	Number of unique proteins	Well-studied interactions	Well-studied unique proteins
**Ito-Core**	834	806	130	165
**Uetz-Screen**	674	797	103	137
**CCSB-YI1**	1799	1271	261	273
**Tarassov**	2761	1122	321	282
**Y2H-Union**	2903	1997	448	463
**HT_Union**	5560	3042	761	1033
**LowBP_LC**	6272	2652	2572	1420

II) Number of interactions and unique proteins in dataset intersections

Additional Table 2
Nuber of interactions in the intersections of individual datasets (*Ito-Core, Uetz-Screen, CCSB-YI1* and *Tarassov*).

**Ito-core**	834				
**Uetz-Screen**	130	674			
**CCSB-YI1**	205	124	1799		
**Tarassov**	45	29	62	2761	
**LowBP-LC**	188	136	291	206	6272

Additional Table 3
Number of unique proteins in the intersections of individual datasets.

**Ito-Core**	806				
**Uetz-Screen**	196	797			
**CCSB-YI1**	260	196	1271		
**Tarassov**	63	47	91	1122	
**LowBP-LC**	273	222	390	240	2652
	**Ito-Core**	**Uetz-Screen**	**CCSB-YI1**	**Tarassov**	**LowBP-LC**

Additional Figure 1

Area-proportional Venn diagrams representing the number of interactions in the intersections between datasets, for union datasets. The diagrams were produced with the online tool http://venndiagram.tk/.

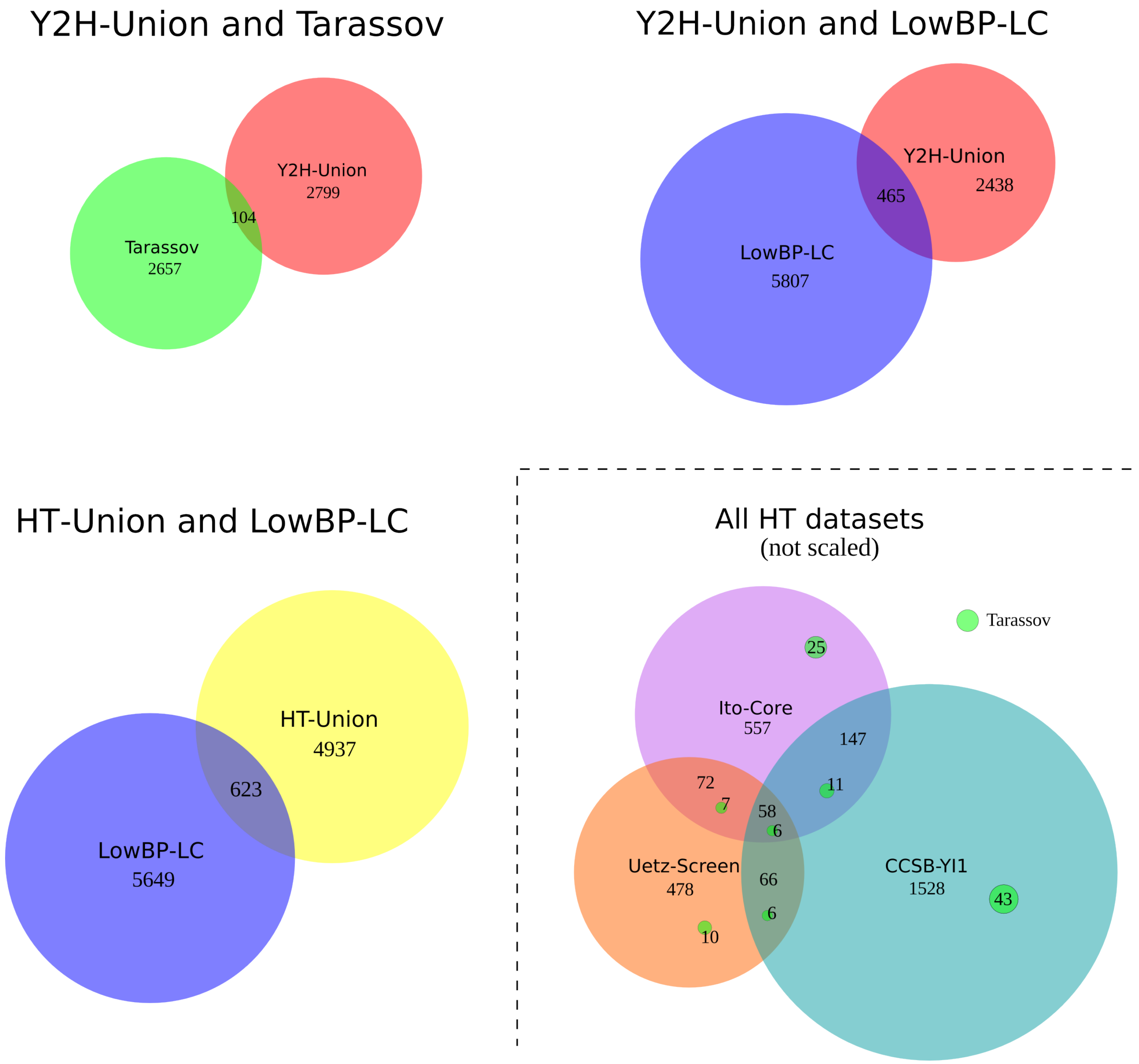

Supplement: Additional file 1 — Number of interactions and proteins in each dataset. Additional file 1 presents the number of interactions and unique proteins in each dataset and intersection of datasets. [file 1471-2105-11-605-S1.PDF]
